# Supplementary material for: EGFR amplification and PI3K pathway mutations identify a subset of breast cancers that synergistically respond to EGFR and PI3K inhibition
Source: Oncogene. 2025 Nov 25;45(1):15–25. doi: 10.1038/s41388-025-03634-3 (PMC12714574; doi:10.1038/s41388-025-03634-3)
Supplement: Supplementary file 1 — Supplementary Figures [file 41388_2025_3634_MOESM1_ESM.docx]

Supplementary Information for

EGFR amplification and PI3K pathway mutations identify a subset of breast cancers that synergistically respond to EGFR and PI3K inhibition

David J. Wisniewski^1^, Donna Voeller^1^, Yonit A. Addissie^1^, Sachin Kumar Deshmukh^2^, Sharon Wu^2^, Maryam B. Lustberg^3^, Darawalee Wangsa^4^, Danny Wangsa^4^, Kerstin Heselmeyer-Haddad^4^, Yoshimi Endo Greer^1^, George W. Sledge Jr.^2^ and *Stanley Lipkowitz^1^

^1^Women’s Malignancies Branch, Center for Cancer Research, National Cancer Institute, Bethesda MD

^2^Caris Life Sciences, Phoenix AZ

^3^Yale Cancer Center, New Haven CT

^4^Genetics Branch, Center for Cancer Research, National Cancer Institute, Bethesda MD

*Corresponding Author (lipkowis@navmed.nci.nih.gov)

This file includes:

Figure S1: Overall survival of patients with EGFR amplification by subtype.

Figure S2: Oncoplot for PI3K pathway alterations in EGFR amplified breast cancer.

Figure S3: EGFR/PI3K dual inhibition significantly reduce downstream signaling in EGFR amplified and PI3K altered TNBC.

Figure S4: Erlotinib treatment does not activate pHER3.

Figure S5: EGFR/PI3K inhibition reduces viability, induces cell cycle arrest in TNBC with EGFR amplification, PI3K alteration.

Figure S6: Dual EGFR/PI3K inhibition induce apoptosis in EGFR amplified and PI3K altered TNBC.


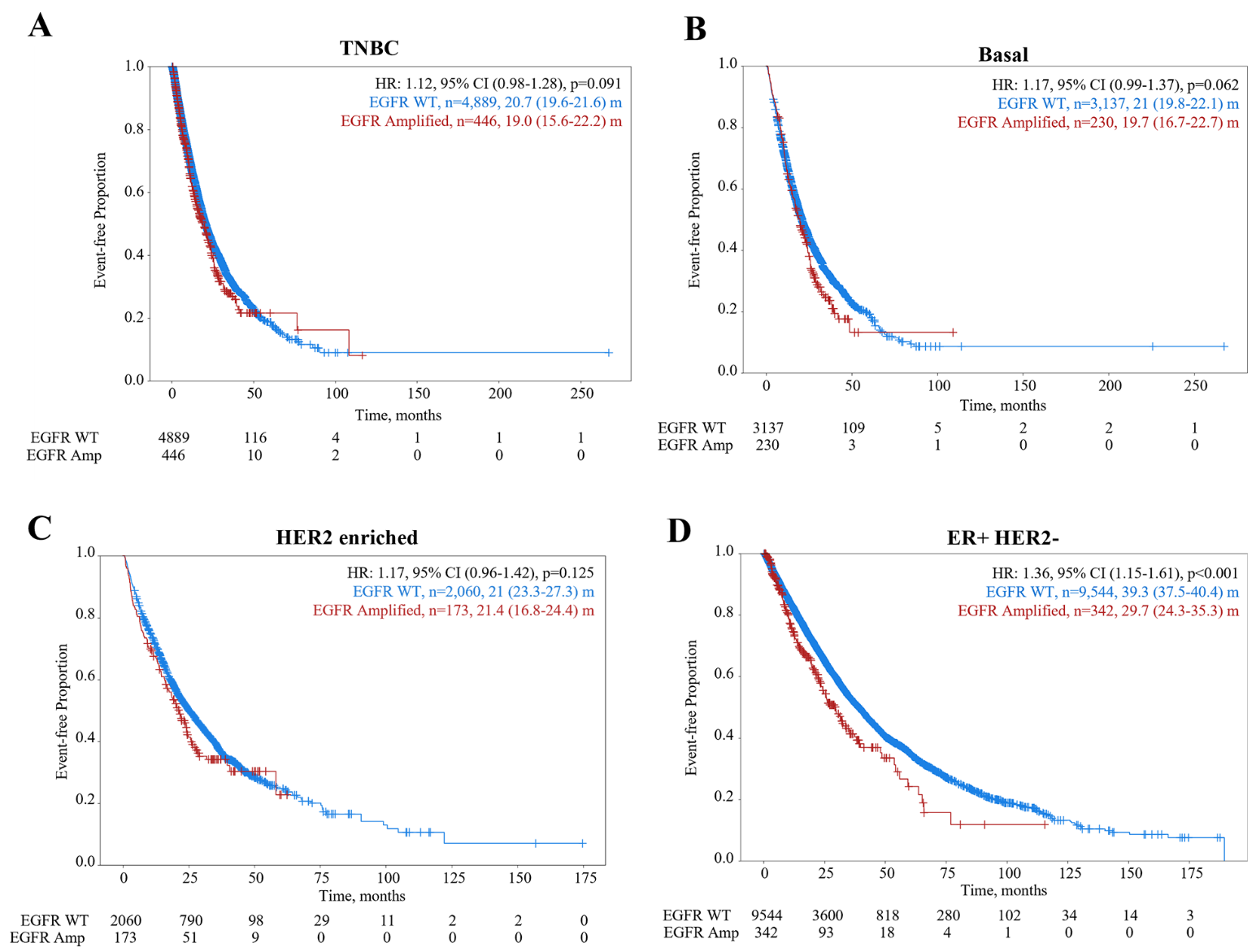


**Figure S1: Overall survival of patients with EGFR amplification by subtype.** Using the Caris dataset, overall survival of breast cancer patients with EGFR amplification (red) vs WT (blue) in A) Triple negative breast cancer, B) Basal, C) HER2 enriched, or D) ER+ HER2-.

**
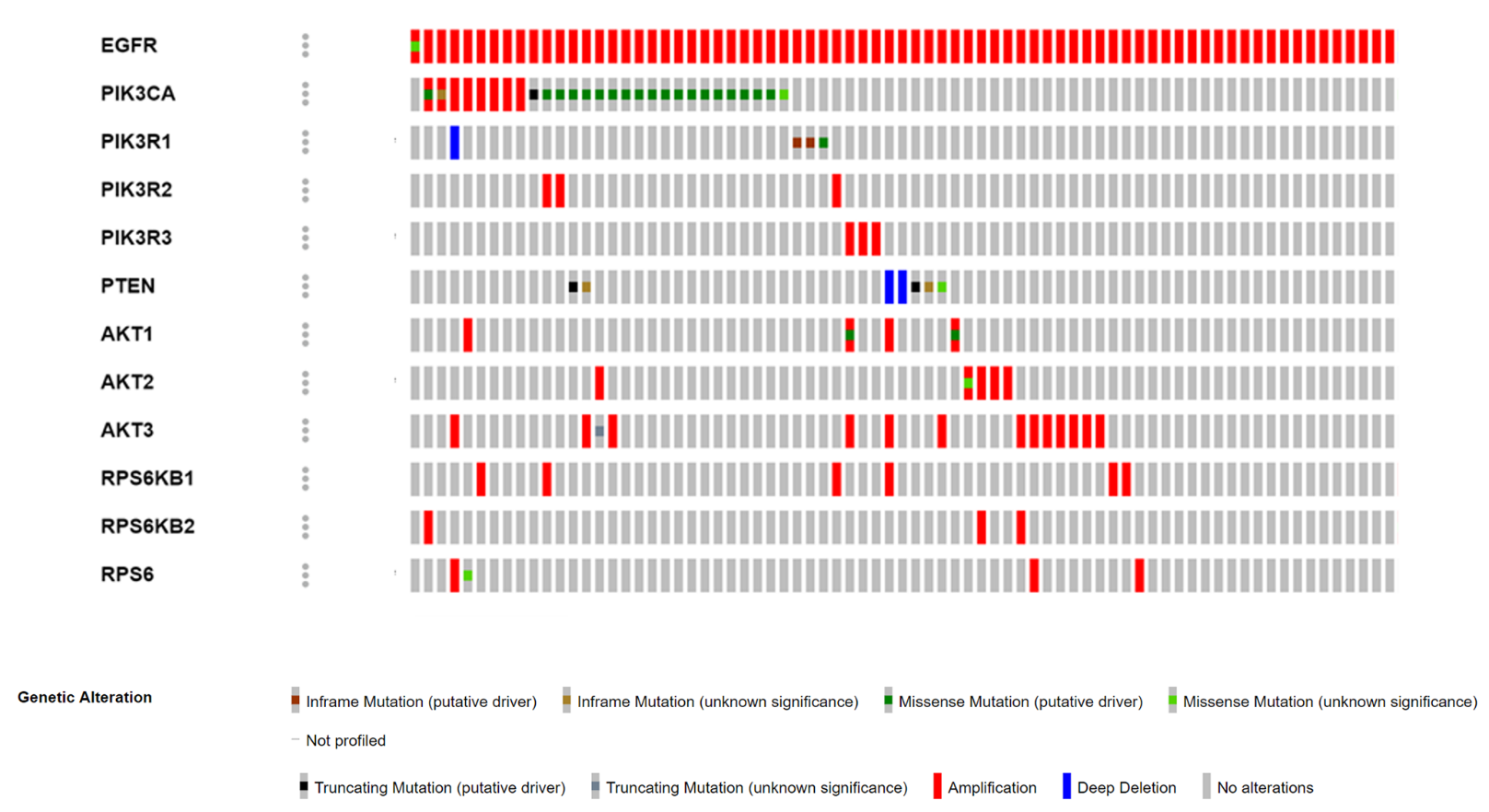
**

**Figure S2: Oncoplot for PI3K pathway alterations in EGFR amplified breast cancer.** Patient data from cbioportal.org plotting varying mutations or amplifications by gene in the PI3K pathway.

**
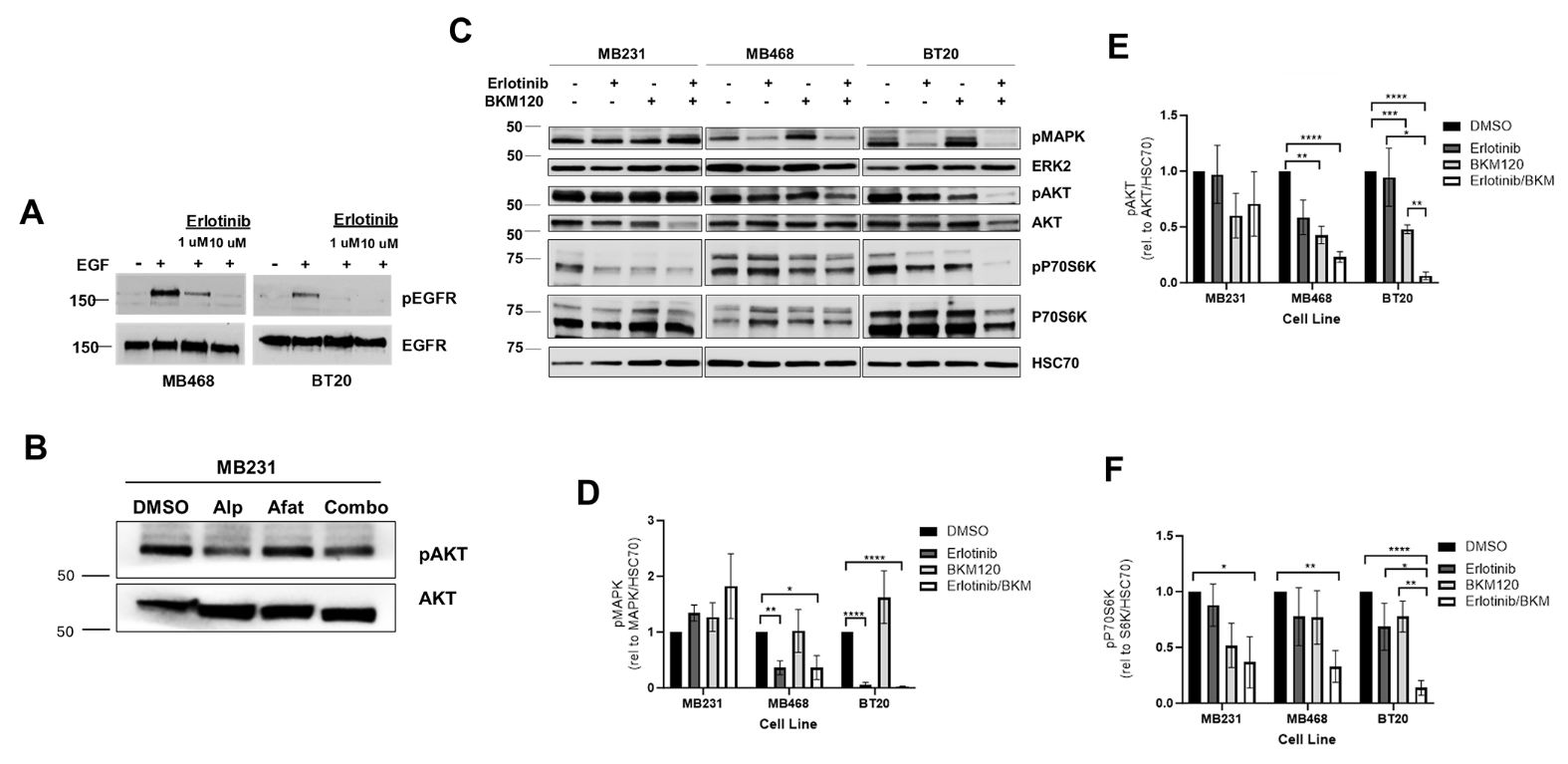
**

**Figure S3: EGFR/PI3K dual inhibition significantly reduce downstream signaling in EGFR amplified and PI3K altered TNBC.** A) MDA-MB-468 and BT20 cells were starved in serum-free RPM1 media for 1 hour with 0, 1 or 10 µM erlotinib, and then stimulated with or without 25 ng/mL EGF for 10 minutes. Cells were lysed and immunoblotted for pEGFR (Y1068) and EGFR (loading control). B) Darker exposure for MDA-MB-231 panel from Figure 4B which was used for quantification. C) MDA-MB-231, MDA-MB-468 and BT20 cells were treated with 10 µM erlotinib, 1 µM BKM120 or a combination of both for 24 hours, and were then lysed and immunoblotted for pAKT (S473), AKT, pMAPK (T202/Y204), ERK2, p-P70S6K (T389), P70S6K and HSC70 (loading control). In three independent experiments, the band density of the phosphorylated protein relative to total protein was averaged ±SEM for D) pMAPK/ERK2, E) pAKT/AKT and F) p-P70S6K/P70S6K. Student’s t-test was performed, where ns indicates not statistically significant, * indicates p<0.05, ** indicates p<0.01, *** indicates p<0.001 and **** indicates p<0.0001.

**
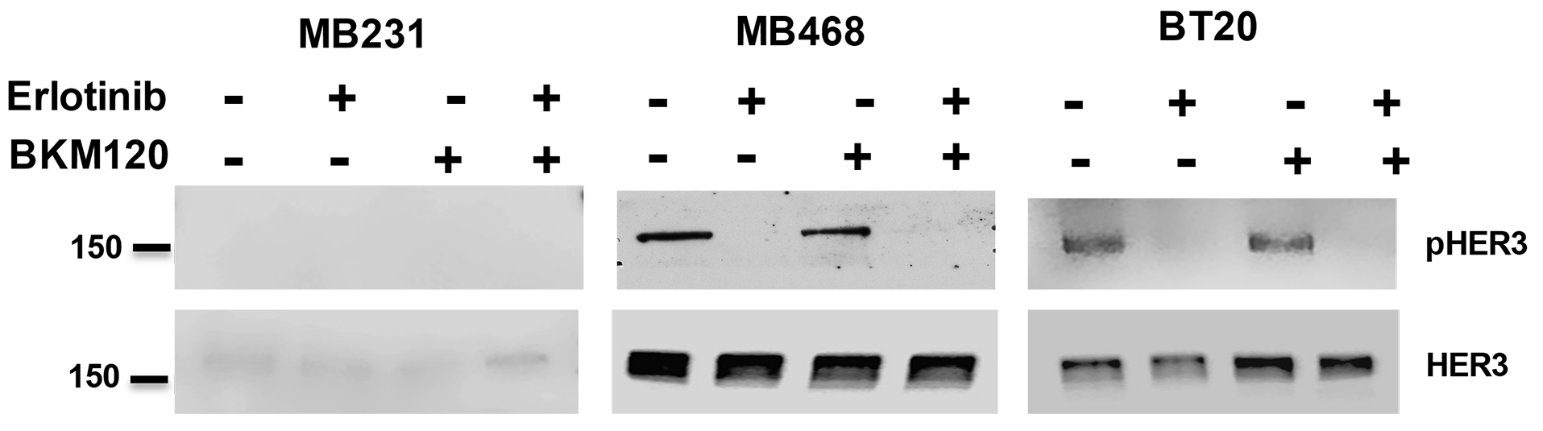
**

**Figure S4: Erlotinib treatment does not activate pHER3.** MDA-MB-231, MDA-MB-468 and BT20 cells were treated with 10 µM erlotinib, 1 µM BKM120 or the combination of both for 24 hours before the cells were lysed and immunoblotted for pHER3 (Y1289) and HER3 (loading control).

**
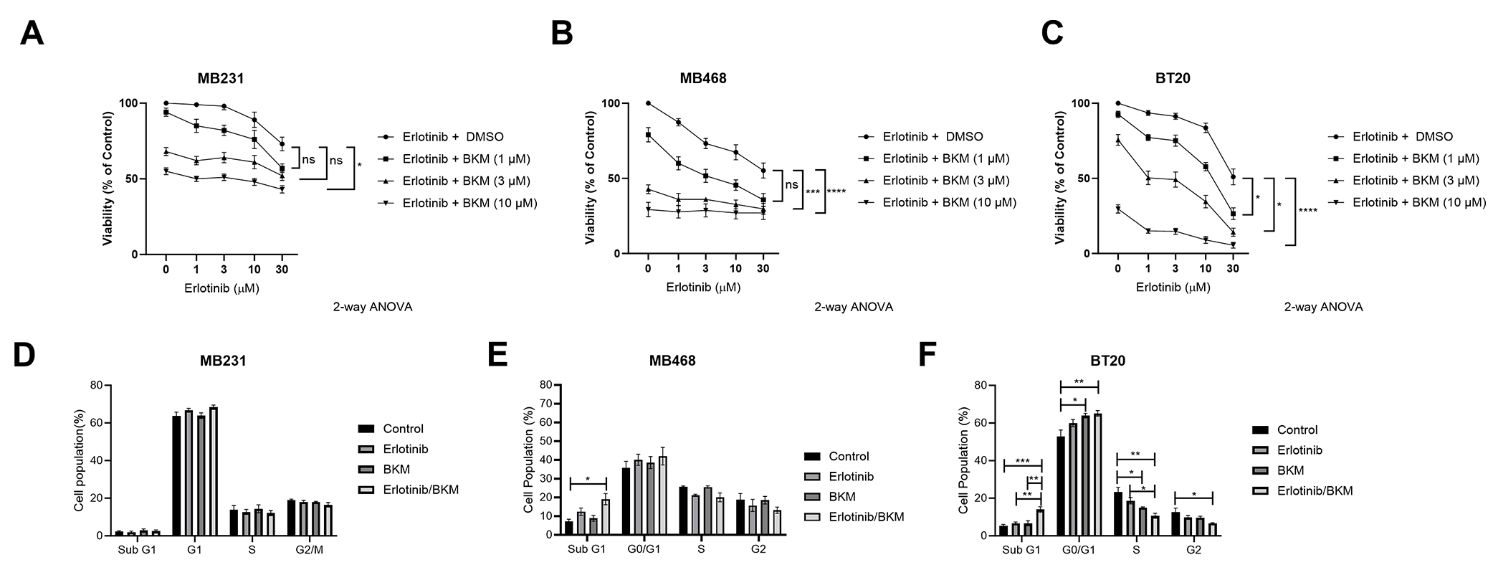
**

**Figure S5: EGFR/PI3K inhibition reduces viability, induces cell cycle arrest in TNBC with EGFR amplification, PI3K alteration.** A) MDA-MB-231, B) MDA-MB-468 and C) BT20 cells were treated with the indicated doses for 48 hours, and then viability was determined by Promega CellTiter-Glo 2.0. Data represents the average ±SEM of three independent experiments, and statistical analysis was performed as Two-Way ANOVA, and the p value indicates interaction, where ns indicates not statistically significant, * indicates p<0.05, *** indicates p<0.001 and **** indicates p<0.0001. D) MDA-MB-231, E) MDA-MB-468 and F) BT20 cells were treated with DMSO control, 1 µM erlotinib or 10 µM BKM120 or the combination of erlotinib and BKM120 for 48 hours, and then were harvested, washed, fixed and stained with propidium iodide before being analyzed by flow cytometry and FlowJo for cell cycle changes. Data represents the average ±SEM of at least three independent experiments, and Two-Way ANOVA statistical analysis was performed where * indicates p<0.05, ** indicates p<0.01 and *** indicates p<0.001.

**
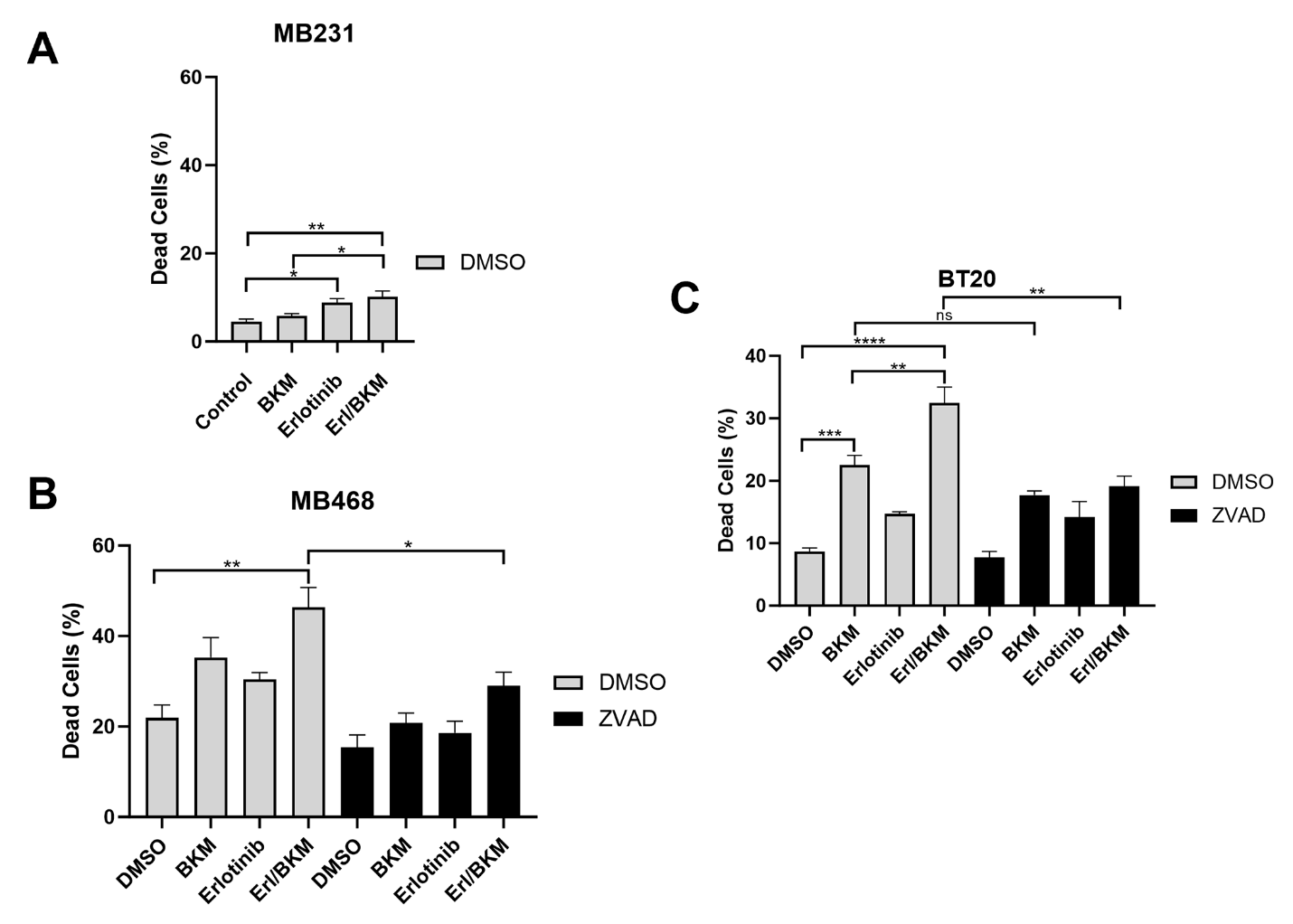
**

**Figure S6: Dual EGFR/PI3K inhibition induce apoptosis in EGFR amplified and PI3K altered TNBC.** A) MDA-MB-231, B) MDA-MB-468 and C) BT20 cells were pretreated with 20 µM Z-VAD-FMK (ZVAD) for one hour, and then were treated with DMSO, 10 µM erlotinib, 1 µM BKM120 or the combination of both, with continued ZVAD treatment for 48 hours. Dead cell percentage was calculated by using CytoTox-Glo by Promega. Data represents the average ±SEM of three independent experiments. Statistical analysis was performed by One-way ANOVA, with ns indicating not statistically significant, * indicating p<0.05, ** indicating p<0.01 and *** indicating p<0.001.
